# Comprehensively addressing postpartum maternal health: a content and image review of commercially available mobile health apps

**DOI:** 10.1186/s12884-021-03785-7

**Published:** 2021-04-20

**Authors:** Laura Tucker, Alan Cuevas Villagomez, Tamar Krishnamurti

**Affiliations:** 1 University of Pittsburgh School of Medicine, 3550 Terrace St, Pittsburgh, PA 15213 USA; 2 Division of General Internal Medicine, University of Pittsburgh, Pittsburgh, PA 15213 USA

**Keywords:** Pregnancy, Postpartum, Peripartum, Maternal health, mHealth, Mobile health, Digital health, Maternal morbidity, Health education, Health disparity, Mobile apps

## Abstract

**Background:**

The United States is currently facing a maternal morbidity and mortality crisis, with the highest rates of any resource-rich nation. In efforts to address this, new guidelines for postpartum care suggest that mobile health (mHealth) apps can help provide complementary clinical support for new mothers during the postpartum period. However, to date no study has evaluated the quality of existing mHealth tools targeted to this time period in terms of sufficiency of maternal health information, inclusivity of people of color, and app usability.

**Methods:**

Preferred Reporting Items for Systematic Reviews and Meta-Analyses (PRISMA) standards were used to review the peripartum apps from the Apple and Google Play stores in either the Health/Fitness, Medical, or Education categories. Apps were evaluated for extent and quality of maternal health information and inclusivity of people of color using an a priori coding scheme. App usability was evaluated using the Mobile Application Rating Scale (MARS) score.

**Results:**

Of the 301 apps from the Apple and Google Play stores, 25 met criteria for final evaluation. Of the 30 maternal health topics coded for, the median number addressed by apps was 19.5 (65%). Peripartum behaviors were more frequently addressed than peripartum outpatient care topics and peripartum acute health risks. The coverage of maternal health information and inclusivity of people of color in app imagery both correlated positively with the MARS usability score of the app. Only 8 apps (32%) portrayed greater than 24% images of people of color- the percent of non-white Americans according to 2019 census estimates. There was no correlation between MARS usability score and number of app users, as estimated by number of ratings for the app available on the app store. In addition, apps with evidence-based maternal health information had greater MARS engagement, information, and aesthetics scores. However, presence of evidence-based information did not correlate with greater numbers of app users.

**Conclusions:**

Current commercially available peripartum apps range widely in quality. Overall current app offerings generally do not provide adequate maternal health information and are not optimally accessible to the target users in terms of inclusivity of women of color or app usability. Apps delivering evidence-based information and more usable design are more likely to meet these standards but are not more likely to be downloaded by users.

**Supplementary Information:**

The online version contains supplementary material available at 10.1186/s12884-021-03785-7.

## Background

The United States is currently facing a maternal morbidity and mortality crisis. The maternal mortality rate has more than doubled since 1990, and currently stands at 17.4 deaths per 100,000 live births-- the highest of any resource-rich nation [1]. The leading documented causes of maternal death are infection, hemorrhage, cardiomyopathy, other cardiovascular conditions, pulmonary embolism, stroke, hypertensive disorders, and amniotic fluid embolism [2]. Newly emerging data also suggests that psychosocial risks, such as intimate partner violence (IPV) and depression-induced suicide, may also play a key role in maternal mortality and morbidity occurring up to a year postpartum [3, 4]. Furthermore, an additional 60,000 U.S. women experience severe maternal morbidity each year [5]. Pregnancy-related maternal mortality and morbidity is disproportionately high among women of color whereby non-Hispanic Black women are more than 3 times more likely to experience pregnancy-related death than non-Hispanic white women [6–8]. There are also disparities in cause of death, with Black women being more likely to suffer from pregnancy-related cardiomyopathy, pulmonary embolism, and hypertensive disorders [9]. In the peripartum period, Black and Hispanic women are at greater risk than white women of both IPV and depression [10–12].

Peripartum health risks can be acute or chronic [13], and confounding symptoms with traditional postpartum experiences can lead to lack of recognition of more severe health risks. For example, while fatigue is a common consequence of postpartum sleep disturbance [14], it may also be an indicator of postpartum depression [15] or cardiomyopathy [16]. A 2018 nine-state maternal mortality review committee demonstrated that an overall lack of knowledge about warning signs and when to receive medical help was one of the most common factors contributing to postpartum maternal mortality [17]. In attempts to more comprehensively reach patients during the postpartum period, guidelines from the American College of Obstetrics and Gynecology (ACOG) have been released suggesting that mHealth apps can help provide supplementary clinical support [18].

Commercial apps communicating about pregnancy-related health, specifically, are becoming increasingly common [19, 20], with healthcare providers endorsing that they play a growing role in maternity care [18, 19]. Using mHealth apps as a widespread form of supplementary clinical support shows promise in both increasing patient education and in narrowing the knowledge gap in health disparate communities [21, 22]. Most women of reproductive age own smartphones, even among the lowest income brackets. Moreover, there is an almost equal distribution of smartphone use across Black, White, and Latino populations [23]. Approximately 80% of smartphone owners use their phones to access health information, and Black and Latino smartphone owners are more likely than White smartphone owners to use their phones to research health conditions [24, 25]. To date, however, there is no existing evaluation of how well commonly-available pregnancy apps explicitly address maternal mortality or morbidity risks or whether the requisite information is presented in a way that makes it accessible to those most at risk.

Currently, the FDA regulates mHealth apps which analyze patient-specific data or give personalized medical guidance but does not regulate apps intended for general patient education [26]. In addition, only a minority of mHealth apps are restricted in access by a prescription from a medical provider or health system [27]. Consequently, many currently-available peripartum mHealth apps have no supervising system ensuring that their published information is accurate and safe for patients.

Even when apps are adequately addressing important health information, their use can be impeded by a failure to tailor them to the specific needs of their audience [28, 29]. Several studies have critically reviewed the content of pregnancy apps [20, 30, 31], with Thomas & Lupton (2016) finding that pregnancy and other reproductive health apps fail to be inclusive of diverse pregnant people. These findings raise concerns for bias in the language and imagery used in peripartum health apps, which risks further distancing health disparate communities. Increasing the diversity of racial representation in health media, on the other hand, may itself work to reduce social disparities in healthcare [32]. Therefore, in order for mHealth apps to most effectively address pregnancy-related health risks, it is crucial that they be actively inclusive and representative of diverse racial and ethnic groups.

Additionally, while a peripartum app may offer comprehensive health information and inclusivity, lack of user-centered app design risks ultimately rendering the app ineffective [33]. An mHealth tool’s usability- defined by Nielsen (2012) as its ease and enjoyability of use [34] is a multifactorial feature promoted by design elements such as interactivity, functionality, and visual appeal [35]. Failure to incorporate these features in app development can lead to outcomes of low goal-achievement and user attrition [36]. The risk of this is even higher among historically marginalized groups, for whom poor mHealth tool usability has the potential to widen already-existing health disparities [37]. Ultimately, without user-centered design , which prioritizes usability, even peripartum apps with high quality information and strong inclusivity may be inaccessible and ineffective for populations who may benefit from the app’s content.

In this analysis, established methods [38, 39] of app review were drawn upon to scope the content of commercially available apps for their ability to adequately inform users about behavioral, outpatient, and acute peripartum risk factors. This was done by determining whether they include the requisite maternal health information to address the leading causes of maternal morbidity and mortality as well as other common postpartum risk factors. Each app was then reviewed for inclusivity in language and imagery and for product usability. These three criteria were employed to understand whether commonly-available apps are sufficient to provide pregnancy-related health information in a way that is accessible to health disparate communities.

## Methods

### Framework

This study employed the Preferred Reporting Items for Systematic Reviews and Meta-Analyses (PRISMA) standards [40], following similar implementation to other mHealth app reviews of content [38, 39].

### Search strategy

The search was limited to the Apple App Store and Google Play, given that 99% of US smartphone users own either Apple or Android products [41]. To comprehensively capture mHealth apps addressing both the pregnancy and postpartum period, a systematic search was conducted using a combination of key terms, reviewed by both obstetric and family medicine providers. These key terms included:

- Pregnancy
- Fetal development
- Fetal growth
- Labor and Delivery
- Postpartum
- Maternal/maternity

The search was limited to apps in the Health/Fitness, Medical, and Education categories (as opposed to, e.g. “entertainment”) to identify those that may contain clinically relevant content.

### Selection criteria

The review was limited to the top 200 apps identified from each search term applied. Apps that met inclusion criteria and were available in both stores were initially evaluated to identify substantial differences in content and imagery across operating systems. If the app showed consistency, the most recently updated app was included in the review.

### Inclusion criteria

Currently available apps (as of August 2019) targeting pregnant patients or containing content for those who have delivered within the preceding 3 months (those in the “4th trimester”) were eligible for review. Apps were required to address education or provide support for physical or mental health needs. For example, apps could be marketed for education, health tracking, medical appointment reminders, or managing stress and anxiety specifically related to pregnancy, childbirth, or early postpartum. To meet those specifications, the inclusion criteria were as follows: (1) apps targeted to pregnant or early postpartum women (up to 3 months postpartum); (2) apps whose primary content was focused on the mother (i.e. not primarily child development or parenting) (2) available through Apple App Store or Google Play; (3) English language; (4) free or paid apps costing less than $10 per app (5) apps available in the following Apple App Store categories: Health & Fitness, and Medical; and (6) apps available in the following Google Play categories: Education, Health & Fitness, Medical.

### Exclusion criteria

Apps were excluded if they were (1) general parenting apps (2) intended for health care professionals; (3) targeted explicitly towards men or partners of pregnant people (4) classified as *e-books* by app store description or reviewers (5) entertainment or social networking apps (6) targeting a single specific symptom or condition that might be experienced in the postpartum period (i.e. cognitive behavioral therapy apps for depression) or (7) required a prescription from a medical professional or health system to fully access the app functions. This last criterion was instated to capture only apps that were available to the general public and not limited by an individual’s access to formal healthcare.

### Screening process

After removal of duplicates, the title and store descriptions of all apps identified in an initial search were screened to determine eligibility for full review. Apps that were eligible for full review were downloaded. For the full review, one primary reviewer evaluated the entire set of Android and iOS apps. The apps were then evaluated independently by one of three secondary reviewers, who were randomly assigned a subset of apps to review. If any uncertainties or disagreements were identified in coding, they were reviewed by the entire research team and resolution was achieved by group consensus.

### Measures

#### Maternal health information

To comprehensively capture information that may be considered necessary for a peripartum app to serve as a clinical support tool, an a priori coding scheme for the apps’ maternal health content was developed based on the primary causes of severe maternal morbidity and mortality and referencing ACOG and CDC guidelines for postpartum care topics [18, 42]. Apps were coded for the presence (or absence) of specific maternal health content and for whether that information was connected to a citation from a professional medical organization or peer-reviewed scientific literature, which was labeled “evidence-based.” Qualitative notes were made of inaccuracies in health information. Table 1 provides details on the 30 specific codes for maternal health information. Elements were separated into three categories: Peripartum Behaviors, Peripartum Outpatient Care, and Peripartum Acute Health Risks.

**Table 1:** Coding scheme for maternal clinical risk information

Maternal Health Information		
Peripartum Behaviors	Peripartum Outpatient Care	Peripartum Acute Health Risks
Breastfeeding/Breast Health	Contraceptive Options	Amniotic Fluid Embolism
Diet/Weight Trajectory	Chronic Health Conditions	Anesthesia Complications
Family Planning	Depression	Cardiovascular/Heart Disease
Infant Safe Sleep	Hypertension	Cerebrovascular Accident/Stroke
Postpartum Infant Care	Intimate Partner Violence	C-Section Complications
Postpartum Weight Loss	Medication Use During Pregnancy	Hemorrhage
Sexual Activity	Medication Use During Breastfeeding	Infections/Sepsis
Sleep Quality	Postpartum Mental Health	Pulmonary Embolism
Smoking	Postpartum Physical Health	Vaginal Birth Complications
	Postpartum Physical/Pelvic Exam	
	Substance Use Disorder	
	Transitioning to Primary Care	

#### Inclusivity

To evaluate inclusivity of the apps, each was coded for references to race in the written content and imagery embedded in the app. All references were coded for the presence or absence of race-based stereotyping and the specific instance was documented with an open-ended note. A second rater then reviewed the instance and note and created a binary code for presence/absence of a biased reference. Definitions for all coding can be found in Supplemental Appendix A. To categorize an app as offering racial inclusivity, it was noted whether at least 24% of app-embedded images displayed non-white presenting individuals. This threshold was chosen to reflect the percentage of the U.S. identifying as a race other than white according to the 2019 US Census estimate [43].

#### Usability

The Mobile Application Rating Scale (MARS) was used to assess the usability of the mHealth apps. This scale consists of 4 subscales: engagement, functionality, aesthetics, and information quality. These subscales are averaged to produce a final mean usability score. Two optional separate subscales are provided to assess subjective quality and perceived impact of the app on user knowledge and behaviors. Each item considered within a subscale uses a 5-point Likert scale (1 = Inadequate, 2 = Poor, 3 = Acceptable, 4 = Good, 5 = Excellent). MARS scores from only the primary rater were used, as this scale has not been validated to incorporate ratings from multiple users in the same score [35].

To assess relative rates of usage in the general public, the total number of Apple and/or Google Play Store ratings for each app was recorded.

### Data analysis

Descriptive statistics, frequencies, correlations, and independent *t* tests were used to summarize the search results and evaluation scores assigned to each app. Data analysis was conducted using IBM SPSS 24.0.

## Results

**Fig. 1:**
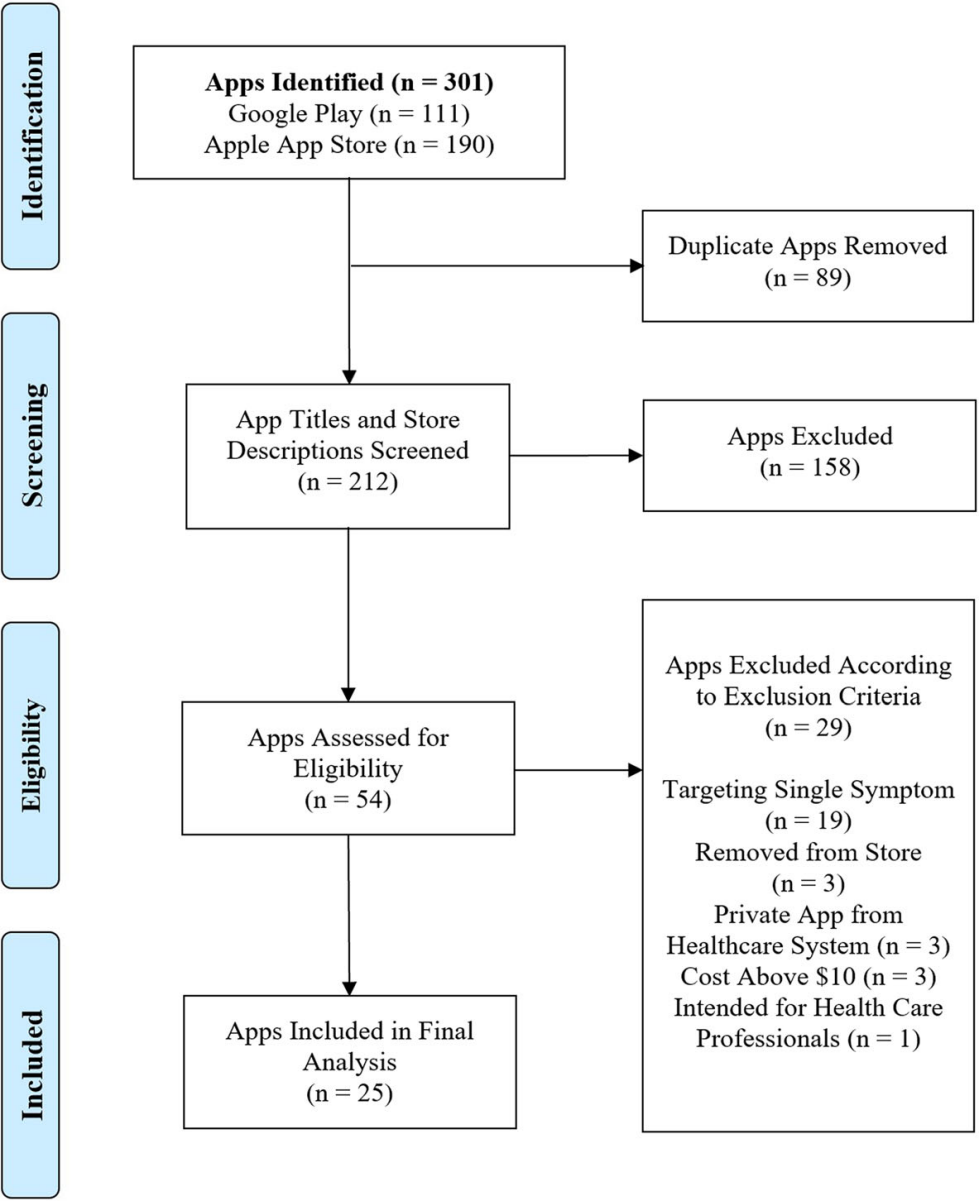
Preferred Reporting Items for Systematic Reviews and Meta-Analyses flowchart of search process for educational mHealth apps with postpartum maternal morbidity and mortality risk content

The PRISMA flowchart of the search process can be seen in Fig. 1. None of the 25 applications coded had received FDA approval.

### Maternal health content

**Fig. 2:**
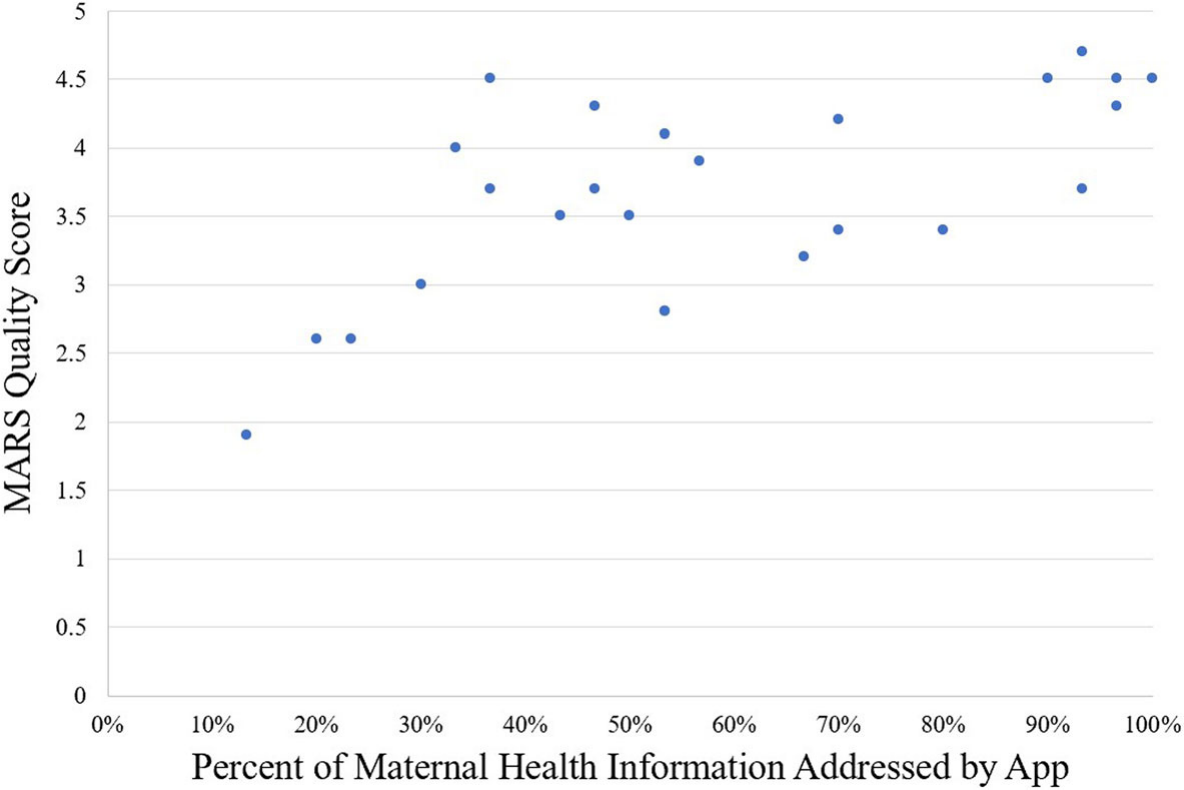
MARS usability score vs percent of maternal health information addressed by app

Of the 25 applications coded, only one, “What to Expect” addressed each of the 30 medical topics included in the Maternal Health coding scheme. The median number of elements addressed by each app was 19.5 (65%), with a range from 4 (13.3%) to 30 (100%). Apps affiliated with a medical organization (i.e. “Circle by Joseph Health” and “Circle by Swedish”) addressed greater than 90% of the peripartum health topics, whereas the remaining, commercially developed, apps almost universally fell below this threshold. The exceptions to this were the commercially developed “WebMD Pregnancy” and “The Bump,” which addressed 29 (96.7%) and 28 (93.3%) of the topics, respectively. A significant (r (23) = 0.643, *p* = 0.001) positive correlation was found between the percent of elements addressed in an app and the app’s MARS score (Fig. 2). Ten of the 25 apps reviewed (40%) used evidence-based information. Examples of information addressing a maternal health topic which was not evidence-based include offering guidance on postpartum depression by explaining that mothers who are “feeling down” are likely feeling so because of dissatisfaction with their bodies, suggesting they should “wear flats,” and “join an online mom group to beat the postpartum blues.”

**Fig. 3:**
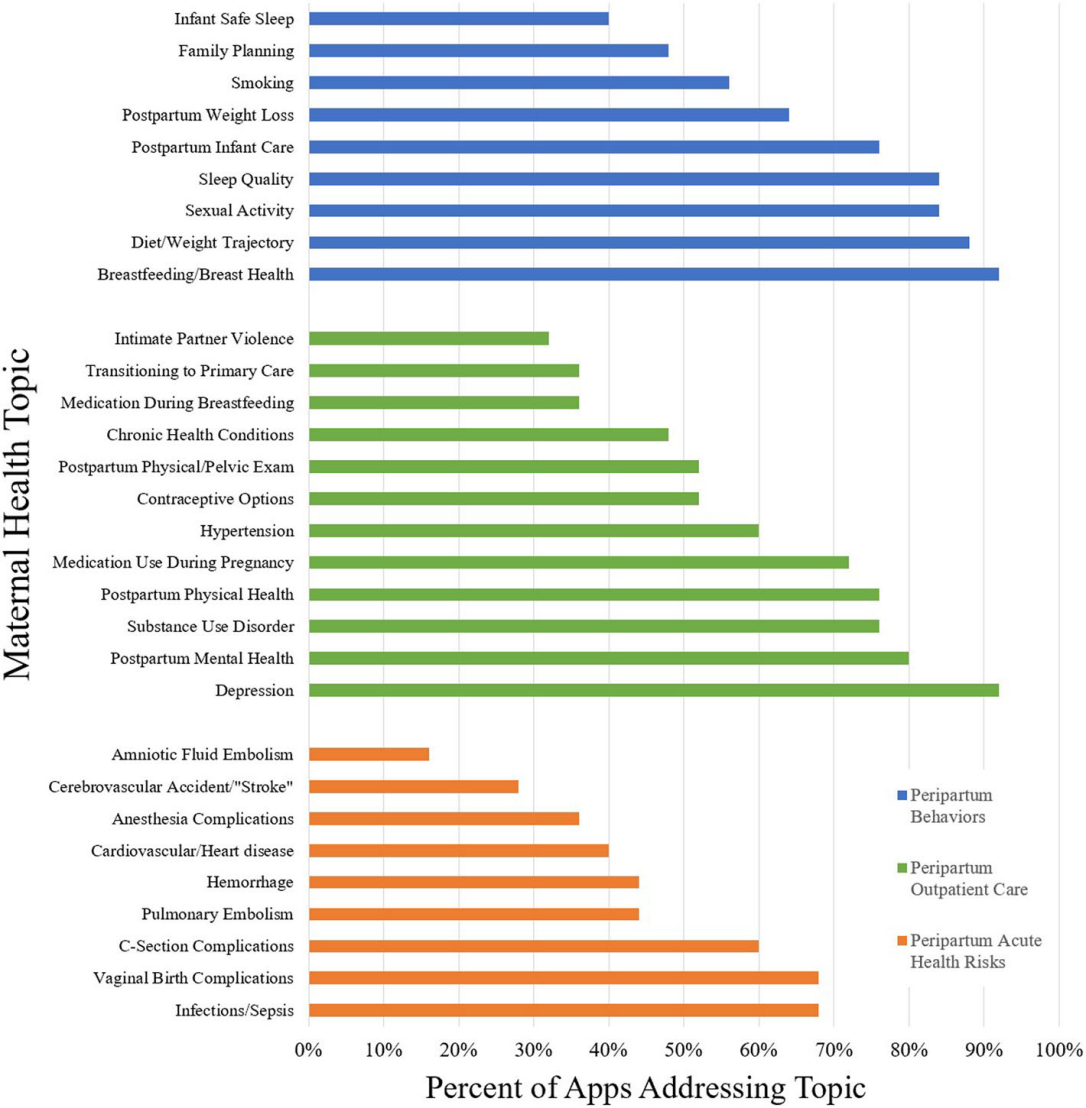
Percent of mobile applications addressing elements of peripartum behaviors, peripartum outpatient care, and peripartum acute health risks

Peripartum behaviors were the most frequently addressed by apps, with 5/9 (56%) addressed by more than 75% of apps in contrast to 4/12 (33%) of peripartum outpatient care topics and 0/9 (0%) of peripartum acute health risks. On average, peripartum behaviors were addressed by 70% of apps, peripartum outpatient care by 59%, and peripartum health risks by 45%. The most widely addressed were breastfeeding/breast health and depression, which were each discussed in 23 apps (92%) (Fig. 3).

### Inclusivity

Only 32% (8/24 apps, one app did not contain images) of applications portrayed greater than 24% of people of color in their imagery. Positive associations were found between the percent of images including people of color and both the MARS score (r (22) = 0.535, *p* = 0.007) and the percent of medical topics addressed (r (23) = 0.756, *p* < 0.001). No significant correlation was found between the number of ratings and percent of people of color (r (23) = 0.271, *p* = 0.20). Of the top-10 most-rated apps, only four (“Pregnancy Tracker-BabyCenter,” “What to Expect,” “The Bump,” and “Ovia Pregnancy Tracker”) reached the 24% diversity threshold. No race-biased language was noted in any of the apps, but 5 apps (20%) were noted to have race-biased imagery in the form of depicting women of color exclusively as patients while white individuals were depicted in diverse roles or in majority healthcare worker roles.

### Usability

The average overall MARS usability score for the 25 apps ranged from 1.9 to 4.7, with a mean score of 3.7, representing an “Acceptable” rating. Overall, apps scored high on functionality (mean = 4.3, SD = 0.5) and lower in engagement (mean = 3.4, SD = 1.1), aesthetics (mean = 3.4, SD = 1.1), information (mean = 3.5, SD = 0.9), and overall usability (mean = 3.7, SD = 0.7). The lowest-scoring MARS subscale was the optional “Subjective Quality,” which is not included in the overall score but takes into account how frequently a user might access the app if relevant to them, whether they would pay for the app, and whether they would recommend it to other users. Subjective quality ratings ranged widely from 1.0–5.0, (mean = 3.0, SD = 1.1). “Circle by Swedish” received the highest overall MARS score, 4.7 (“Good”), followed by “What to Expect,” “Pregnancy Tracker-BabyCenter,” “WebMD Pregnancy,” and “Track Pregnancy&Baby:Preglife,” each with a rating of 4.5 (“Good”). None of the apps received an overall score of “Excellent.”

No significant correlation was found between the applications’ number of store ratings (a proxy for prevalence and popularity) and the proportion of the medical topics the app addressed (r (23) = 0.252, *p* = 0.224) or the MARS score (r (23) = 0.255,*p* = 0.219). While the most-rated app, “Pregnancy Tracker-BabyCenter” (993,267 total ratings), addressed 90.3% of medical topics and received a MARS score of 4.5 (“Good”), the next-most rated application, “Pregnancy +” (685,310 ratings) addressed only 48.39% and received a MARS score of only 3.5 (“Acceptable”). “Circle by Swedish,” which addressed 96.7% of medical topics and received the highest MARS score of the apps in this review at 4.7 (“Good”), only received 1216 ratings.

Apps with evidence-based information showed a significantly greater mean percent of maternal health information addressed in the app (t (20) = 4.02, *p* = 0.001). These apps also had higher overall MARS scores, (t (23) = 4.74, *p* < 0.001), with higher ratings on the engagement (t (23) = 4.06, *p* < 0.001), aesthetics (t (17) = 3.10, *p* = 0.007), and information sub-scores (t (23) = 5.09, *p* < 0.001). A significant difference was not observed in the MARS functionality score between the two groups of apps (t (21) = 1.15, *p* = 0.265). There was also no significant difference in the number of store ratings between the apps whose information were and were not evidence-based.

### Highest-quality apps

Four apps met or exceeded all of the following criteria for an acceptable peripartum mHealth application: 1) Shared evidence-based information; 2) addressed greater than or equal to 90% of maternal health information; 3) imagery portrayed at least 24% people of color; 4) received a MARS score of at least 4 (“Good”) in each subcategory of engagement, functionality, aesthetics, and information; 5) did not depict race-biased imagery or language. These apps were “What to Expect,” “Pregnancy Tracker - BabyCenter,” “Circle by Swedish,” and “Circle by Joseph Health.”

## Discussion

Updated ACOG guidelines have promoted the use of mobile app-based support in postpartum care [18]. This review has shown that while some peripartum apps provide the medical information, inclusivity, and usability that is optimal for a successful clinical support tool, there is wide variability among the currently available products and many apps fail to meet these standards. The most frequently downloaded apps do not necessarily communicate the most peripartum health information and are not more likely to share evidence-based information. Lack of patient knowledge of warning signs is known to be a leading contributor to postpartum hemorrhage, cardiovascular disease, and cardiomyopathy- three of the leading causes of postpartum morbidity and mortality in the US [17]. The absence of any reference to these specific health risks in over half of the apps reviewed suggests that current offerings are not adequate to provide critical information to those who seek it.

Peripartum behavior and outpatient health topics were addressed more frequently than acute mortality and morbidity risks. For example, breastfeeding health information was provided by almost every app. This highlights an area where the current mobile apps can well serve postpartum women, the majority of whom have difficulty breastfeeding and for whom access to education provides measurable benefits [44, 45]. The content covered may be in response to market demand from app users, given that peripartum women have also expressed desire for additional education regarding postpartum depression, contraception, and physical well-being [46], each of which were addressed in a majority of the apps. However, the lack of evidence-based information in most apps gives pause to recommending them for medical information, exemplified by the app whose mental health discussion only addresses only postpartum blues and recommends only online support groups. While social support online and in person can be protective for postpartum depression [47, 48], there is a striking increased risk of mortality and morbidity for women who do not receive professional medical and psychological treatment [49]. Connecting patients with apps which minimize or trivialize mental health information may inadvertently put them at greater risk of negative consequences.

The peripartum mHealth apps also tended to lack inclusivity of women of color- and while more inclusive apps were associated with higher usability, they were not more likely to be downloaded by users. One notable shortfall in inclusivity was seen in the rates of maternal health information provided by the apps. Peripartum infections and mental health concerns, conditions that are more prevalent among non-Hispanic white women [17], were addressed by most of the apps, while embolism and cardiomyopathy, which disproportionately affect women of color, were infrequently addressed. A digital divide currently exists in the US, with Black citizens being less likely to have a desktop or laptop and more likely to prefer using mobile phones to seek health information [24]. As apps are a major form of information delivery on smartphones, the subtle bias in providing health information that disproportionately aids white women risks widening the health literacy and therefore peripartum mortality gap. Moreover, a majority of the peripartum apps were shown to lack inclusivity of women of color in their imagery. Black patients are more likely to prefer providers of the same race, [50, 51] and culturally appropriate information has been endorsed as a method to increase health equity in the reproductive health of Black women [52]. The subset of apps which represent women of color only as patients also introduces potential damage, as increased racial minority representation in health professions has been shown to reduce health disparities [53]. Providers should be aware that using the currently available commercial apps as clinical support tools may not be inclusive of and effective for women of color.

Even when peripartum apps contain evidence-based, comprehensive health information and are inclusive of marginalized groups, they remain ineffective if they fail to deliver a highly usable mHealth product. In this review there was no correlation between and app’s usability and number of app store ratings (and thus by extension the number of users the apps reached). Smartphone users are more likely to download an app they perceive as popular, associating popularity with product quality [54]. In this saturated market of peripartum mHealth tools, consumers may therefore be drawn to download apps of worse informational quality and usability simply because they possess 100-fold more app store ratings than some higher-quality apps. Importantly, however, actual adoption and employment of mHealth technology is directly related to a tool’s usability and perceived usefulness rather than its popularity [55]. In this review, the apps collectively received an overall only “Acceptable” MARS usability score on subscales of engagement, aesthetics, and information quality. This general lack of user-centered design among peripartum mHealth products and relative popularity of less-usable apps is currently a risk to patients relying on these tools for health information. Access to evidence-based medicine via a mobile app has important potential to improve health behaviors of peripartum women [31, 56], but poor usability promotes attrition from mHealth tools and thus diminishes the potential effectiveness of the health information being delivered [36]. In addition, lack of user-centered design supporting mHealth tools makes engagement more challenging and thus further marginalizes populations who already experience a digital divide in healthcare [57]. Therefore, while there is an abundance of commonly available peripartum apps, their overall limited usability risks harm by both reducing effectiveness for users and further widening the gap between health disparate groups.

While mobile apps offer a promising opportunity to improve peripartum care, the current commonly available options are not yet optimal for this purpose. In order for mHealth apps to act as a true complement to maternal healthcare, they must deliver evidence-based and comprehensive information in a manner that is both inclusive of women of color and highly usable [58]. Among a broad market of peripartum mHealth tools, a few products exist which achieve these standards; however, these are vastly outnumbered by apps which exclude important health information, alienate health disparate groups, and fail to provide the user-centered design that is an essential contributor to app efficacy. Healthcare providers should be aware of the dearth of high quality peripartum apps and should consider the components comprising a high-quality tool when discussing mHealth app use with their peripartum patients.

## Limitations

In limiting this review to commercially available products, mHealth apps that have been developed specifically by health systems for their patient populations were unable to be included. While these types of apps will likely share evidence-based information and capture postpartum health risks more comprehensively, they may also have more limited reach. Second, the intent of these apps may not explicitly be to serve as a clinical support tool, though this study surveys whether these tools could fit this purpose. The intent of women using these tools and whether they think of them as clinical support is also not known, and merits exploration in future work. A third limitation to this study, and all studies evaluating diversity of images, is the risk of mis-categorizing images of Black and other women of color. Having multiple reviewers make these judgments aimed to minimize this risk; however, it cannot be certain that the researchers’ categorization is consistent with how the individual in the image would self-identify. Similar limitations apply to discriminatory text and language. While a subjective measure, the evaluation by multiple reviewers and research group discussion of language attempted to mitigate these limitations. Finally, while the MARS scale is an accepted and validated measure, it was employed only by the researchers and not by the population using the app and, as such, the research team’s judgments may not fully reflect those of the population.

## Conclusion

While ACOG has recommended that mHealth apps can provide clinical support to patients during the postpartum period, no comprehensive review has before been done of the information, inclusivity, and usability of the currently available products. This review has found that many popular commercially available peripartum apps do not provide adequate maternal health information, are not inclusive of women of color, and are not optimally usable for patients. Clinicians should be aware of these deficiencies and can use shared decision making to aid patients in finding apps which are accurately informative and without harmful biases or exclusion. Four applications were ultimately found to be acceptable by the criteria of this review, but application content is constantly evolving. While providers may endorse the products highlighted in this review to their patients, general recommendation of apps developed by health systems (e.g. “Circle by Swedish”) may be the most appropriate solution to ensuring quality information delivery to patients amidst a dynamic peripartum app landscape.

## Supplementary Information

**Additional file 1: Supplemental Appendix A.** Inclusivity Coding Definitions

## Data Availability

The datasets used and analyzed during the current study are available on Open Science Framework.

## Abbreviations

ACOG: American College of Obstetricians and Gynecologists
CDC: Center for Disease Control and Prevention
MARS: Mobile Application Rating Scale
PRISMA: Preferred Reporting Items for Systematic Reviews and Meta-Analyses
