# Supplementary material for: Comprehensively addressing postpartum maternal health: a content and image review of commercially available mobile health apps
Source: BMC Pregnancy Childbirth. 2021 Apr 20;21:311. doi: 10.1186/s12884-021-03785-7 (PMC8059182; doi:10.1186/s12884-021-03785-7)
Supplement: Supplementary file 1 — Additional file 1: Supplemental Appendix A. Inclusivity Coding Definitions [file 12884_2021_3785_MOESM1_ESM.docx]

Supplemental Appendix A: Inclusivity Coding Definitions

| **Title** | **Definition** |
| --- | --- |
| **Number of Images Viewed** | Number of individuals portrayed in imagery embedded in app content, not including individuals displayed in advertisements. |
| **Number of Images of People of Color** | Number of individuals portrayed in imagery embedded in app content who visually appear to not be Caucasian, not including individuals displayed in advertisements. |
| **Qualitative Assessment of Language Used for Racism** | Note absence or presence of language embedded in the app which demonstrates discrimination, prejudice, or bias against an individual or group on the basis of race. Include written example. |
| **Qualitative Assessment of Images Used for Racism** | Note absence or presence of imagery embedded in the app which demonstrates discrimination, prejudice, or bias against an individual or group on the basis of race. Include written example. |
